# A Structure-Aware Convolutional Neural Network for Automatic Diagnosis of Fungal Keratitis with In Vivo Confocal Microscopy Images

**DOI:** 10.1007/s10278-021-00549-9

**Published:** 2023-04-04

**Authors:** Shanshan Liang, Jing Zhong, Hongwei Zeng, Peixun Zhong, Saiqun Li, Huijun Liu, Jin Yuan

**Affiliations:** 1 State Key Laboratory of Ophthalmology, Guangdong Provincial Key Lab of Ophthalmology and Visual Science, Zhongshan Ophthalmic Center, Sun Yat-Sen University, Guangzhou, Guangdong China; 2 National Innovation Center for Advanced Medical Devices, Shenzhen, Guangdong China; 3 School of Electronics and Information Technology, Sun Yat-Sen University, Guangzhou, Guangdong China

**Keywords:** Fungal keratitis, Confocal microscopy cornea imaging, Automatic diagnosis, Convolutional neural network

## Abstract

Fungal keratitis (FK) is a common and severe corneal disease, which is widely spread in tropical and subtropical areas. Early diagnosis and treatment are vital for patients, with confocal microscopy cornea imaging being one of the most effective methods for the diagnosis of FK. However, most cases are currently diagnosed by the subjective judgment of ophthalmologists, which is time-consuming and heavily depends on the experience of the ophthalmologists. In this paper, we introduce a novel structure-aware automatic diagnosis algorithm based on deep convolutional neural networks for the accurate diagnosis of FK. Specifically, a two-stream convolutional network is deployed, combining GoogLeNet and VGGNet, which are two commonly used networks in computer vision architectures. The main stream is used for feature extraction of the input image, while the auxiliary stream is used for feature discrimination and enhancement of the hyphae structure. Then, the features are combined by concatenating the channel dimension to obtain the final output, i.e., normal or abnormal. The results showed that the proposed method achieved accuracy, sensitivity, and specificity of 97.73%, 97.02%, and 98.54%, respectively. These results suggest that the proposed neural network could be a promising computer-aided FK diagnosis solution.

## Introduction

Fungal keratitis (FK) is one of the most severe vision-threatening ocular diseases worldwide, especially in developing countries, and seriously affects the patients’ quality of life. More than 70 kinds of opportunistic fungi have been demonstrated to cause FK, and these are mainly divided into filamentous fungi and yeast-like fungi [1]. The accurate diagnosis and early treatment are crucial to avoid severe complications, such as corneal perforation, hypopyon, endophthalmitis, and even blindness [2]. The current commonly used clinical examination methods include slit lamp examination, corneal scraping microscopy, fungal culture, tissue biopsy, KOH test, PCR, and confocal microscopy [2]. However, they all have limitations: (a) a slit lamp examination can observe only the surface of the cornea and can provide only a simple initial diagnosis based on symptoms [3]; (b) scrape microscopy, tissue biopsy, and the KOH test can damage the cornea [4]; (c) fungal culture takes approximately 1 week to develop and makes timely diagnosis difficult [5]; and (d) the cost of PCR is too high to be suitable for widespread clinical diagnosis [6].

Confocal microscopy has been used for the early diagnosis of FK, and the results until now have been promising. However, this diagnosis is mainly depending on the subjective experience of ophthalmologists or experts, which is time-consuming and has a high false positives rate [2]. Furthermore, diagnosis based on confocal microscopy is not able to distinguish the species and the activity of fungi, while it is not able to perform quantitative analysis and quantify the fungal hyphal number. Therefore, there exists a great need for an automatic classification system that can recognize hyphae. However, there have been limited studies so far aiming to overcome this limitation. The data acquisition of in vivo confocal images with fungal hyphae is difficult and time-consuming, while the images may show a complex structure, which makes accurate diagnosis difficult. Experienced ophthalmologists are required for the collection and labeling of the data to solve the first problem. Moreover, machine learning methods are deployed to tackle the second problem.

The standard processing firstly involves the manual extraction of proper features and then their utilization to train a support vector machine model to perform image classification [7]. Since the features are extracted manually, the whole classification system is transparent and interpretable. However, the hand-crafting process also limits the flexibility and refrains the design of general features. Deep-learning-based methods, which can automatically extract features from the original images without requiring hand-crafted features, have been recently applied and greatly improved the performance of many computer vision tasks. In general, in deep-learning-based methods, a convolutional neural network is trained on a large dataset automatically extracting hierarchical features. Harnessing the power of deep learning methods, automatic diagnosis of FK based on neural networks has also undergone extensive development [2, 8]. However, training a single network may not fully exploit the structural features of images in the case of FK classification. In the present research work, we suggest incorporating prior knowledge into the training of neural networks to substantially exploit the structural information of images and facilitate strong classification performance. Specifically, a two-stream convolutional neural network is deployed combining two commonly used network architectures in computer vision, GooLeNet [9] and VGGNet [10]. For the one stream, the network is used to directly extract features from the input image. For the second stream, the possible structure of fungal hyphae is first extracted to be used as prior knowledge and then the processed image is used as the input of the stream. Finally, the features extracted by the two streams are integrated to be used as inputs for the prediction models. The intuition is that the pixel intensity in regions with fungal hyphae is generally higher than the pixel intensity in other regions. Accordingly, the mean of all pixel values is subtracted to extract the possible structure of fungal hyphae as prior knowledge. This two-stream convolutional neural network was named as structure-aware convolutional neural network (SACNN).

## Related Work

### Deep Learning on Image Classification

Since AlexNet [11] was first applied to ImageNet classification [12], the computer vision community has witnessed a rapid development of deep learning (a.k.a. deep neural networks). The performance on image classification and other computer vision tasks has greatly improved due to the advantages of deep learning [9, 10, 13, 14]. The deep convolutional neural networks, which are typically used in computer vision tasks, generally consist of several sequential convolutional layers; they are optionally followed by nonlinear function and pooling operations and followed by several fully connected layers, with their overall topology exhibiting tremendous potential in image-related tasks. The AlexNet, overcame other non-neural-network-based methods by large margins, demonstrating state-of-the-art performance on large-scale datasets [11]. The network in this tool consists of five convolutional layers and three fully connected layers, where each convolutional layer is followed by ReLU nonlinearity [15] and normalization called local response normalization. Data augmentation and dropout [16] were exploited in the training of the neural network to prevent overfitting [11]. However, although there are only five convolutional layers and three fully connected layers, the computational complexity of the network is high, due to the large kernel in the convolutional layers, which may prevent the network from increasing its depth. The recently introduced networks, such as VGGNet [10] and GoogLeNet inception v1 [9], attempt to use small kernels to lower the computational complexity while maintaining the same receptive field of the input image in an attempt to overcome the latter problem. Specifically, for VGGNet, the size of the convolution kernel is set to 3 × 3. Compared with the 3 × 3 pooling kernels of AlexNet, VGGNet has 2 × 2 pooling kernels. In addition, larger numbers of layers and channels (i.e., deeper and wider networks) are used in VGGNet. Generally, deeper networks result in larger model capacity and can learn more powerful and discriminative representations. For GoogLeNet inception v1, multibranch convolution is designed to extract image features from different convolution kernels. The network is also extended to be deeper and wider to improve its performance. More details about those state-of-the-art models can be found in their original papers [9, 10, 17, 18]. In this paper, we mainly utilize VGGNet [10] and GoogLeNet inception v1 [9] to classify medical images.

### Automatic Diagnosis of Fungal Keratitis

The accurate and quick diagnosis of FK is of great clinical significance, as microbial culture takes approximately 7 days, and confocal imaging has obtained the potential to achieve automatic diagnosis with the recent advances in image processing techniques using artificial intelligence. In 2016, Qiu et al. developed an automatic diagnosis method based on local binary patterns and support vector machines [19]. This method achieved an accuracy of 93.53% on a dataset with approximately 200 images [19]. Wu et al. proposed an adaptive robust binary pattern as a better texture descriptor [20] to further improve the performance, and the accuracy improved to 99.74% on a dataset with approximately 400 images [20]. However, these methods are based on traditional image processing methods, and the datasets were relatively small, resulting in a limited role in clinical applications. With the rapid development of deep learning in computer vision applications, it has become natural to apply deep-learning-based methods to the automatic diagnosis of FK. Liu et al. proposed applying convolutional neural networks combined with image preprocessing algorithms to diagnose and classify fungal hyphae, achieving high accuracy of 99.95% [21]. They used data augmentation and image fusion to preprocess the images to improve the classification performance. Specifically, they first augmented images by image rollovers, then proposed sub-area contrast stretching to preprocess the images, and third fused the preprocessed images with the original images using a histogram matching fusion algorithm [21]. This combination of image preprocessing and convolutional neural networks presented satisfying results. However, the proposed image preprocessing is slightly complex, making it too time consuming for real-time processing in clinical applications. In addition, Lv et al. recently adopted ResNet for the automatic diagnosis of FK [22]. The proposed method in this study fuses prior knowledge to improve the classification performance and utilizes a larger dataset to train the network compared to the aforementioned comparative studies. Additionally, elaborate comparison experiments with previously proposed methods were conducted in the present study.

## Method

**Fig. 1:**
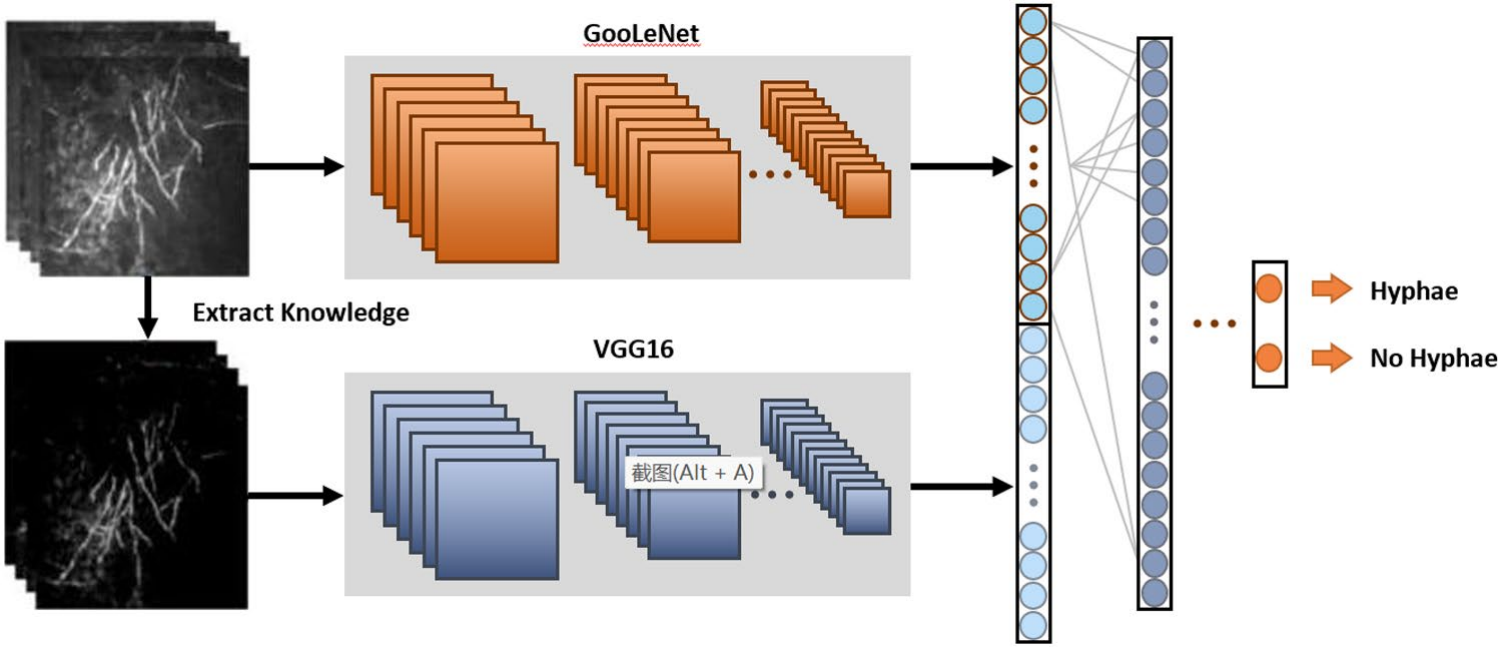
The overall proposed framework of the two-stream structure-aware convolutional neural network

The aim of this research work was to distinguish confocal corneal images from fungal hyphae using images without fungal hyphae and deep-learning-based methods. In general, a typical method to address the problem is to train a convolutional neural network with a classification loss function. In this paper, however, we suggest incorporating simple but effective prior knowledge to further improve the classification performance. For the confocal corneal images with fungal hyphae, the pixel intensity of the region with fungal hyphae is generally higher than the one in the region without fungal hyphae. Accordingly, we can extract the approximate structure of hyphae to assist the classification. In this paper, the mean of all pixel values is subtracted to extract the structure of the hyphae, with this method being simple but effective. The extracted approximate structure is used as prior knowledge to improve the prediction performance. For this purpose, a two-stream convolutional neural network, called SACNN, is used. SACNN consists of the main stream extracting image-level features and an auxiliary stream extracting prior-level features. The main stream processes the original image to extract hierarchical features, and the auxiliary stream processes the corresponding prior knowledge (i.e., approximate structure of hyphae). The overall framework is presented in Fig. 1. Regarding feature extraction of prior knowledge, it is expected that the auxiliary stream can learn discriminating features and enhancing the feature of hyphae structure for images with fungal hyphae, which may improve the classification accuracy. Then, the features extracted by the two streams are further integrated to perform the final prediction. For each stream, commonly used networks in computer vision applications were adopted, i.e., Inception v1 [9] for the main stream and VGGNet [10] for the auxiliary stream. Other networks, such as ResNet [13] and DenseNet [18], may also be used as the two streams. However, the main focus of this paper was to incorporate prior knowledge on the structure of fungal hyphae to improve the classification performance, and thus, less emphasis was given on optimizing the selection of network architectures and only commonly used and high-performing architectures in other computer vision applications were utilized.

## Experiments

### Dataset and Implementation Details

The dataset used in the system is the SYSU_OC_ Keratis2019 dataset, which consists of 7278 confocal images capturing the cornea that has been collected from FK patients at the Zhongshan Ophthalmic Center, Sun Yat-sen University, from November 2015 to May 2019. Among these patients, 3862 had hyphae and 3416 did not. All images were produced with an HRT3-CM confocal laser cornea microscope from Heidelberg Company in Germany, and there were no selection criteria for age and sex. The study protocol was approved by a properly constituted institutional review board (Zhongshan Ophthalmic Centre ethics committee of Sun Yat-sen University, Guangzhou, China), and the study was conducted under the ethical principles of the Declaration of Helsinki (2017KYPJ104).

The confocal images of FK were captured as follows. A sterile Tomocap (Heidelberg Engineering GmbH, Dossenheim, Germany) was mounted over the objective of the microscope (Zeiss, × 63), and polyacrylic acid 0.2% (Viscotears, Novartis) was used as a coupling agent between the cap and the lens objective. The options for image acquisition included section (a single image at a particular depth), volume (a series of images over 60-μm depth), and sequence scans (a video sequence at a particular depth). The wavelength of the laser, which was employed in the HRT II/RCM, was 670 nm, and each standard 2-dimensional image consists of 384 × 384 pixels covering an area of 400 µm × 400 µm.

The image preparation process firstly starts with the confirmation of the diagnosis of FK by the results of the corneal microbial culture of the fungi. Then, the IVCM images were randomly assigned to three junior corneal experts for initial screening and labeling. Each expert reviewed a set of images, and the other two corneal experts were invited to confirm the labeling results. If the diagnoses of the first- and second-round experts were inconsistent, the image was being submitted to the highest level of corneal expertise to obtain a final decision. A total of 3862 images with hyphae were selected. Finally, all the images with hyphae were traced by the drawing board with a red brush of 1-pixel width in the computer.

The total dataset is further split into a training and a test set, respectively. Twenty percent of the total dataset was selected as the test set, which consisted of 1455 images, with 683 of them being of patients without fungal hyphae and 772 of them coming from patients with hyphae.

**Fig. 2:**
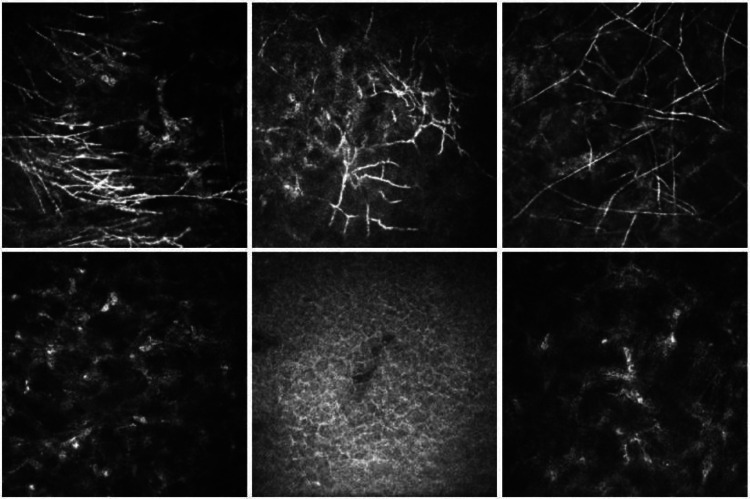
Examples of images with and without fungal hyphae. Top row: with fungal hyphae. Bottom row: without fungal hyphae

**Fig. 3:**
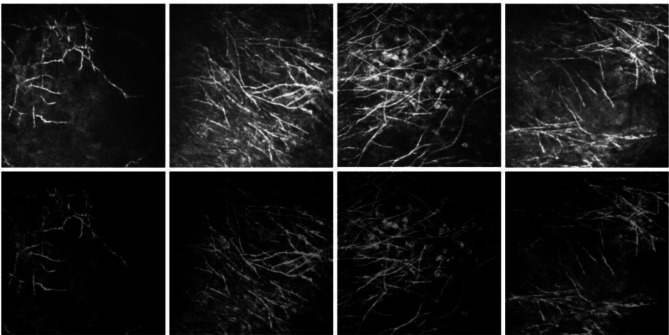
Examples of original images and corresponding prior images. Top row: original images. Bottom row: prior images

Some examples in the dataset are shown in Fig. 2. Examples of prior images containing possible hyphal structures are shown in Fig. 3.

The size of the images was resized and fixed to 224 × 224, and these processed images were used to train the network. The initial learning rate was 0.0001, decreasing linearly during the training process. Kaiming initialization [23] was used to assist the network training for the initialization of the network weights and biases. The batch size was set to 8 to limit the usage of GPU memory. In addition, commonly used binary cross-entropy loss was utilized as the loss function and the Adam optimizer [24] was deployed to update the network parameters. The network training terminated after 100 epochs.

### Experimental Results

**Fig. 4:**
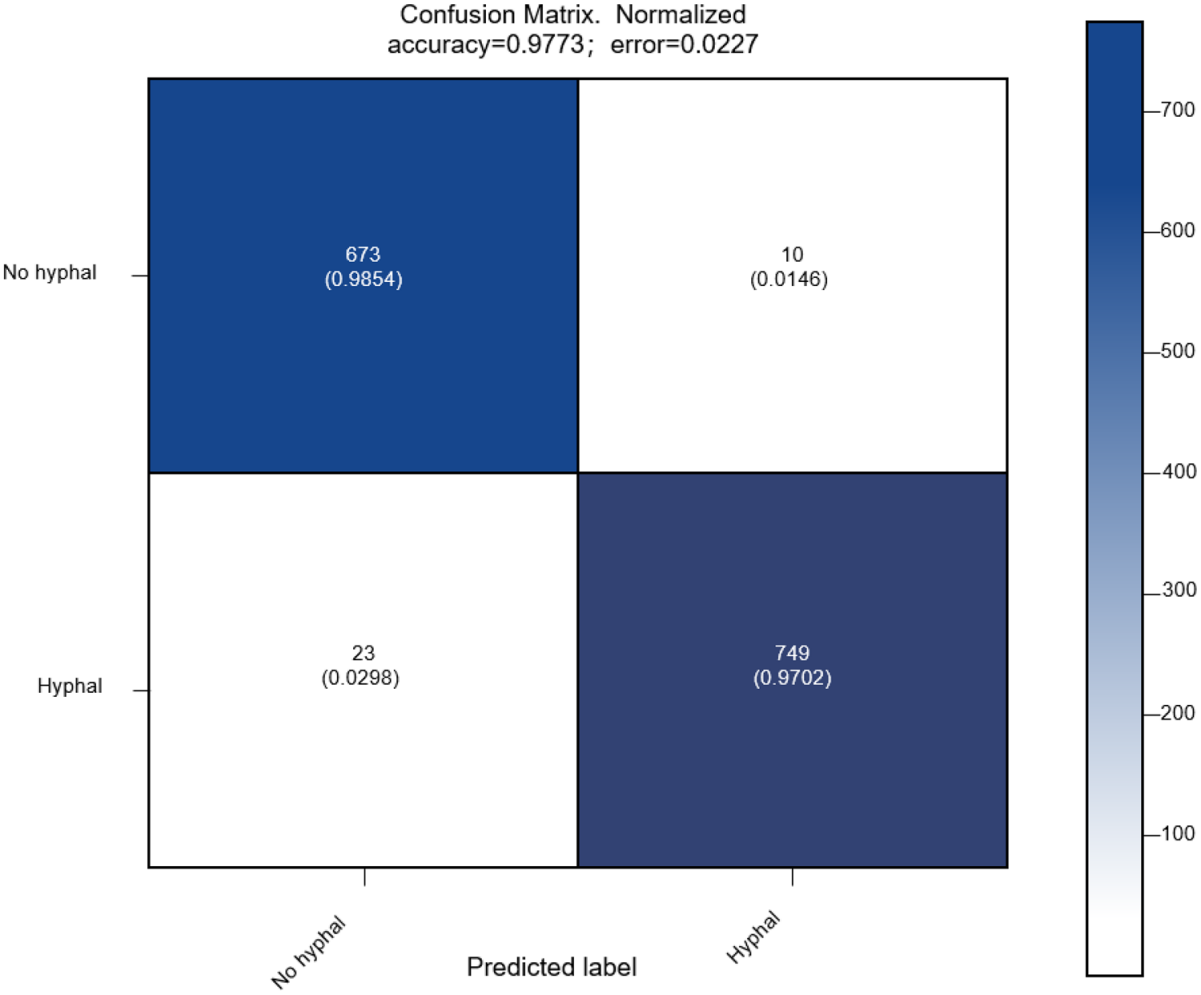
Confusion matrix of the classification results on the test set

The trained model was evaluated on a test set consisting of 1455 images. The confusion matrix of the classification results is shown in Fig. 4. According to these results, 10 images without fungal hyphae were diagnosed as fungal images, and 23 images with fungal hyphae were diagnosed as nonfungal images. Several statistical indexes, including accuracy, precision, sensitivity, specificity, F1-score, area under the ROC curve (denoted as ROC-AUC), and area under the precision-recall curve (denoted as PR-AUC), were calculated to quantitatively evaluate the performance of the proposed method. These metrics are defined as follows:

1 $${\text{Accuracy}} = \frac{{{\text{TP}}\;{ + }\;{\text{TN}}}}{{{\text{TP}}\;{ + }\;{\text{TN}}\;{ + }\;{\text{FP}}\;{ + }\;{\text{FN}}}}$$

2 $$\Pr {\text{ecision}} = \frac{{{\text{TP}}}}{{{\text{TP}}\;{ + }\;{\text{FP}}}}$$

3 $${\text{Sensitivity}} = \operatorname{Re} {\text{call}} = \frac{{{\text{TP}}}}{{{\text{TP}}\;{ + }\;{\text{FN}}}}$$

4 $${\text{Specificity}} = \frac{{{\text{TN}}}}{{{\text{TN}}\;{ + }\;{\text{FP}}}}$$

5 $$F1 = \frac{{2 \times \Pr {\text{ecision}}\; \times \;\operatorname{Re} {\text{call}}}}{{\Pr {\text{ecision}}\; + \;\operatorname{Re} {\text{call}}}}$$

where TP is the number of true positive samples, TN is the number of true negative samples, FP is the number of false-positive samples, and FN is the number of false-negative samples. The positive samples here are images with fungal hyphae.

**Fig. 5:**
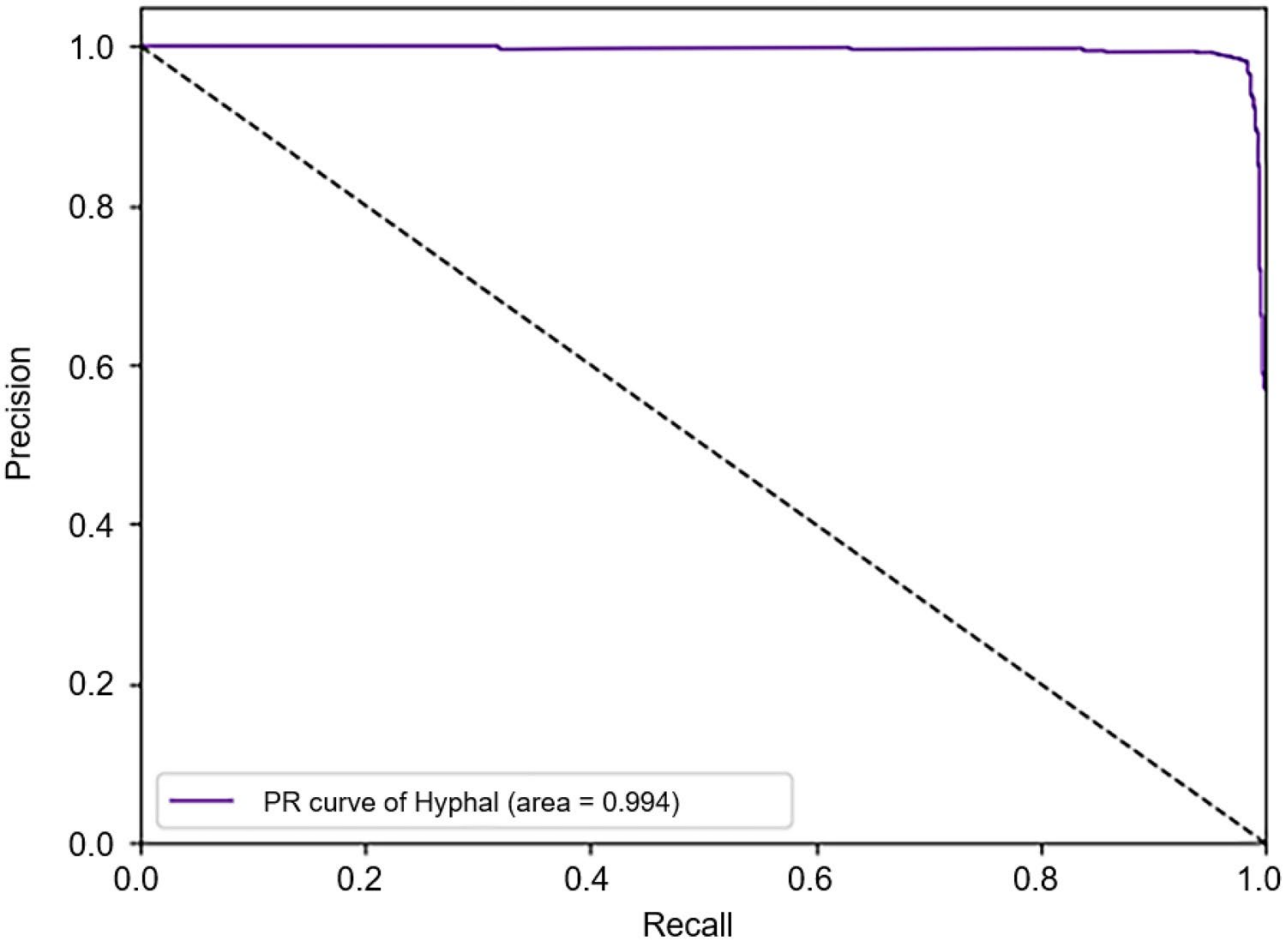
Precision-recall curve of the classification results on the test set

**Fig. 6:**
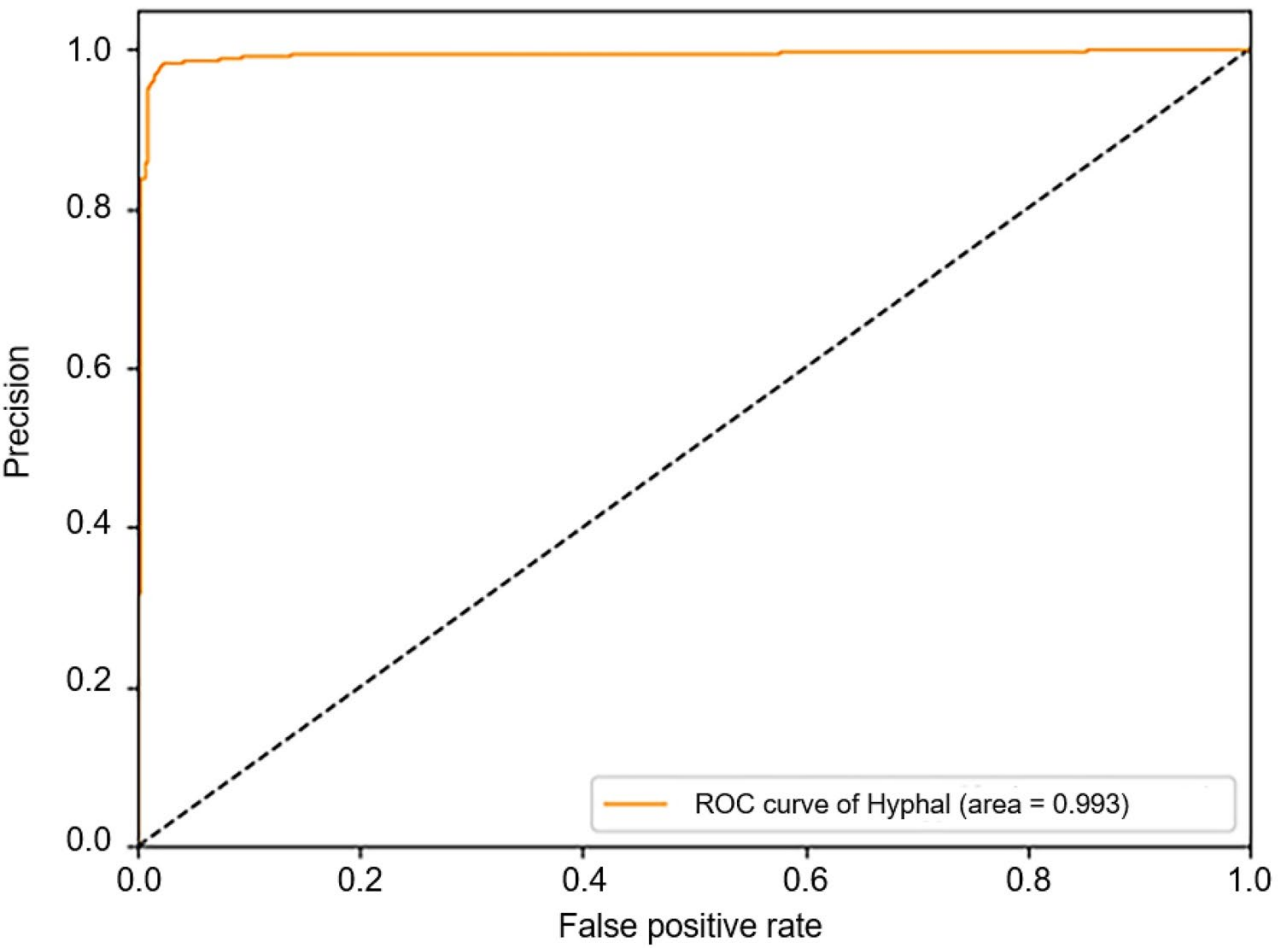
ROC curve of the classification results on the test set

The accuracy, precision, sensitivity, specificity, and F1-score were 0.9773, 0.9868, 0.9702, 0.9854, and 0.9784, respectively. The precision-recall and the ROC curves are shown in Figs. 5 and 6, respectively. The proposed method was benchmarked against previously proposed algorithms in [21] to further confirm the improvement in its classification performance. The method proposed in [21] was used to train neural networks using the training set of this study. Experimental results on the same training and test sets are shown in Table 1. The results indicate that our method can achieve better performance.

**Table 1:** Comparison results of the proposed and other existing methods

Method	Accuracy	Precision	Sensitivity	Specificity	F1-score	ROC-AUC	PR -AUC
ALEXNET + HMF	0.7237	0.9920	0.4831	0.9956	0.6498	0.9650	0.9720
GOOGLENET + HMF	0.9567	0.9810	0.9365	0.9795	0.9582	0.9880	0.9880
SACNN	0.9773	0.9868	0.9702	0.9854	0.9784	0.9930	0.9940

An ablation study was also conducted to validate the effectiveness of the fusion of prior knowledge. Firstly, the auxiliary stream was removed and only the main stream was trained (i.e., GoogLeNet [9]) to perform the prediction. Secondly, the main stream is removed and only the auxiliary stream was trained (i.e., VGGNet [10]) to make the prediction. The results are shown in Table 2. The results showed that the fusion of prior knowledge is indeed beneficial for the classification performance.

**Table 2:** Ablation results

Method	Accuracy	Precision	Sensitivity	Specificity	F1-score	ROC-AUC	PR-AUC
GOOGLENET	0.9677	0.9714	0.9676	0.9678	0.9695	0.9930	0.9950
VGG16	0.8158	0.8310	0.8223	0.8225	0.8255	0.9130	0.9170
SACNN	0.9773	0.9868	0.9702	0.9854	0.9784	0.9930	0.9940

## Conclusions

In this paper, we propose the incorporation of simple but effective prior knowledge to classification models of FK. The mean of all pixel values was subtracted from every image to extract the approximate hyphae structure as prior knowledge. To incorporate this prior knowledge, a two-stream convolutional neural network is utilized to facilitate the fusion. The main stream can extract image-level features from the original images. For the auxiliary stream, the network is expected to learn conducting feature discrimination and enhancement of images with fungal hyphae. The experimental results demonstrated that the proposed method can achieve higher accuracy compared to other existing methods.

An interesting future direction could be to incorporate and fuse more complicated prior knowledge, especially domain knowledge in the medical field, to further improve the diagnosis accuracy. Moreover, it is worth exploring the combination of deep-learning-based methods and domain knowledge. However, interpretability is very important in medical applications, and this is a limitation of existing deep-learning-based methods. In future research, we aim to design more interpretable algorithms, further supporting physicians to make clinical decisions.

## Data Availability

The datasets generated and/or analyzed in the present study are available upon a reasonable request to the corresponding. This manuscript includes all the available data in this study.
